# Supplementary material for: Impaired Release of Neutrophil Extracellular Traps and Anemia-Associated T Cell Deficiency in Hereditary Hemorrhagic Telangiectasia
Source: J Clin Med. 2020 Mar 12;9(3):767. doi: 10.3390/jcm9030767 (PMC7141391; doi:10.3390/jcm9030767)
Supplement: Supplementary file 1 [file jcm-09-00767-s001.pdf]

Supplemental material

Table S1. Immune parameters in relation to the self-assessed course of the disease.

		**Worse** (n = 51)	**Better or the same** (n = 18)	***p***
**Lymphocytes**	%	22 (17–28)	23 (15–28)	*0.76*
	10^6/mL	1.3 (0.9–1.7)	1.1 (0.8–1.6)	*0.26*
**T lymphocytes**	%	60 (54–69)	57 (49–64)	*0.19*
	10^6/mL	0.8 (0.6–-1.2)	0.6 (0.5–0.8)	*0.10*

n = number of patients. Data are presented as median (25-75 percentiles). Mann-Whitney U test was used for comparison of two independent groups.
